# Treatment options in autism with epilepsy

**DOI:** 10.3389/frcha.2024.1265081

**Published:** 2024-02-22

**Authors:** Alejandro Cano-Villagrasa, Francisco J. Moya-Faz, Nadia Porcar-Gozalbo, Miguel López-Zamora

**Affiliations:** ^1^ UCAM Universidad Católica de Murcia, Murcia, Spain; ^2^ Facultad de Ciencias de la Salud, Universidad Internacional de Valencia (VIU), Valencia, Spain; ^3^ Departamento de Psicología Evolutiva y de la Educación, Facultad de Psicología y Logopedia, Universidad de Málaga, Málaga, Spain

**Keywords:** ASD, epilepsy, surgical treatment, children, pharmacotherapy

## 1 Introduction

Autism Spectrum Disorder (ASD) is a neurodevelopmental disorder characterized by deficits in social communication, repetitive behaviors, and restricted interests (1). ASD is associated with a broad range of concomitant conditions, with epilepsy being one of the most significant and frequent (2, 3). Although ASD and epilepsy exhibit different symptoms and underlying causes (4), there is a strong relationship in their physical and cognitive manifestations (4, 5), as confirmed by the fact that up to 30% of individuals with ASD may have epilepsy (6).

In a recent meta-analysis, a comprehensive exploration of seventy-three studies delved into the point prevalence of active epilepsy, ultimately distilling 67 estimates from 63 unique studies for inclusion in the meta-analysis (6, 7). The resultant synthesis revealed a pooled point prevalence of active epilepsy standing at 6.38 per 1,000 persons (7). Shifting focus to the USA, several population-based studies in the pediatric realm uncovered a notably higher incidence of epilepsy within the first year of life, ranging from 82.1 to 118 per 100,000 person-years, eclipsing the rate observed in older children, which hovered around 46 per 100,000 person-years. An additional prospective study mirrored this trend, recording an incidence of 75 per 100,000 live births before 6 months and 62 per 100,000 between 6 and 12 months—an unprecedented revelation that diverges significantly from previously published estimates (8). Beyond point prevalence, twelve studies contributed insights into the annual prevalence of active epilepsy (9). The pooled outcome showcased an annual prevalence of 2.83 per 1,000 persons, while the median annual prevalence charted at 3.91 per 1,000 persons (10). Nestled within this complex tapestry of prevalence, the pediatric population grappling with both ASD and epilepsy emerges as a multifaceted, multidimensional conundrum. This intricate landscape unfolds against a backdrop of diverse symptoms, genetic foundations, and clinical expressions (11). ASD and epilepsy embark on an evolutionary journey from childhood to adulthood, casting a growing therapeutic demand that resonates not only with patients and their families but reverberates within educational systems and society at large (11, 12).

Delving into the realm of seizures, their classification unfolds into episodes characterized by either motor or non-motor symptoms—a spectrum encompassing generalized tonic-clonic seizures, clonic seizures, tonic seizures, and myoclonic seizures (13–15). Typical absences manifest with a sudden onset, interrupting ongoing activities, featuring a fixed gaze, unresponsiveness to speech, and a duration spanning seconds to half a minute, culminating in a swift recovery (16). Crucially, it's paramount to underscore that the term “absence” doesn't equate to a fixed gaze, as this characteristic may also manifest in focal onset seizures (17). In contrast, atypical absence displays more pronounced changes in tone compared to typical absence, marked by a non-abrupt onset or termination. Myoclonic absence introduces a sudden, brief (<100 ms), involuntary, non-repetitive, and non-sustained contraction with associated absence. Adding another layer to this intricate spectrum, absence with eyelid myoclonia involves eyelid jerks occurring at a frequency of less than 3 per second, often with eyes deviated upward, typically lasting less than 10 s. This phenomenon is frequently triggered by eye closure and bears a high likelihood of photosensitivity (18).

In 2017, the ILAE Epilepsy Classification unfurled three diagnostic levels (15), enriching our understanding of this intricate landscape. The first level takes root in the seizure type, encompassing an expansive array of concepts—be they focal, generalized, or of unknown onset. This level also introduces two pivotal concepts woven into the diagnostic fabric: comorbidity (associated pathological entities) and etiology. Some patients may find a resting place at this diagnostic level due to investigational limitations, either as a valid endpoint or as the precursor in their diagnostic journey (19). Stepping into the second diagnostic level, the scene unfolds when at least one EEG and brain imaging study graces the diagnostic landscape. Here, the focus shifts to determining the type of epilepsy—focal, generalized, combined (merging both focal and generalized seizures, a common theme in various epileptic syndromes), or of unknown origin. Etiological diagnoses cast their net into structural, genetic, infectious, metabolic, immune, or unknown categories (20). In the intricate dance of medical complexities, a patient may wear more than one etiological hat. For instance, a patient with tuberous sclerosis might proudly display cortical tubers, embodying both a structural and genetic etiology (21). The final frontier, the third diagnostic level, immerses us in the realm of epileptic syndromes—a composite tapestry weaving together common characteristics such as seizure types, specific EEG findings, age-dependent imaging features, onset and remission age, specific triggering factors, daily variations, prognosis nuances, and distinctive intellectual and psychiatric comorbidities. It's a canvas where etiological and treatment implications unfurl their tales (22, 23). This paradigm-shifting classification boldly swaps out the term “benign” for the more apt descriptors “self-limited” or “drug-responsive.” The unfolding narrative beckons further exploration and inquiry into the ever-evolving lexicon of epilepsy classification.

Regarding ASD, there is significant interindividual clinical variability. Within families, it has been indicated that both genetic and environmental factors influence the phenotypic expression of ASD (12, 13). Many of the core or nuclear symptoms, while reduced to deficits in social communication and interaction and repetitive or stereotyped behaviors, do not manifest their extensive symptomatic expression (10–13).

The neural pathways proposed as the biological basis in ASD associated with epilepsy are related to: (1) imbalance between the glutamatergic (excitatory) and GABAergic (inhibitory) pathways; (2) alteration in the cholinergic pathway; and (3) disruption in synaptic plasticity and oxidative stress (23).

ASD and epilepsy are associated with a high level of psychiatric comorbidity, including anxiety, self or hetero-aggression, depression, hyperactivity, obsessive-compulsive disorder, attention difficulties, tics, eating disorders, executive function impairment, and sleep disorders, which may require pharmacological therapy at some stage of evolution, as well as other relevant symptoms (24), highlighted in the following Table 1:

**Table 1:** Comorbidities associated with ASD or epilepsy condition.

Disorders/Comorbidities	ASD (%)	Epilepsy (%)
Anxiety disorder	10–80	64
Sensory processing disorder	>80	20–35
Sleep disorder	40–80	2.7
Attention deficit Hyperactivity disorder	30–80	4.4
Oppositional defiant disorder	20–80	1.7
Intellectual disability	20–60	1.8–4.1
Obsessive-compulsive disorder	10–40	12
Depression	10–30	2.1
Tics	10–20	-

Adapted from Popow et al. (24) and Boesen et al. (25).

There are commonly other comorbidities related to severe neurological alterations. A well-studied example of which association between ASD and epilepsy is tuberous sclerosis, in which infantile epileptic spasms appear to be a risk factor for ASD regardless of the location and number of brain tubers (26). All of this is related to the fact that, during the ontogenesis of the nervous system, certain brain areas mature chronologically before others, following a genetically determined program (27). If this maturation process is interfered with by frequent seizures, the consequences can be serious for the consolidation of emerging cognitive functions and the development of the social brain (28). Moreover, epileptiform discharges can occur in the absence of clinical seizures but still impact the brain's maturation process (29). Therefore, tuberous sclerosis represents one of the most attractive etiopathogenic models—genetic, biochemical, structural, and neurophysiological—to understand the bidirectional interaction between ASD and epilepsy (27).

Considering this information, the main objective of this opinion article is to discuss and update the primary pharmacological and surgical treatments for the care of the pediatric population diagnosed with ASD and epilepsy.

## 2 Pharmacological treatment in the pediatric population with ASD and epilepsy

The pharmacological treatment choice in the pediatric population with ASD and epilepsy is complex and requires an interdisciplinary approach. Specialists in neurology, pediatrics, and psychiatry must design a personalized treatment plan to address the child's needs (28).

As mentioned earlier, the comorbidity of ASD and epilepsy presents a wide range of associated symptoms in individuals (29). The pharmacological care for this population becomes significantly complex. Many experts recommend treatment plan begins with the management of ASD-related symptoms using medications that help reduce or alleviate these symptoms (30). Several studies refer to the administration of drugs such as antipsychotics or antidepressants to address the semiological characteristics that appear in this disorder (31). Table 2 provides a list of the most common alterations in ASD and their pharmacological treatment.

**Table 2:** Pharmacological Treatment of symptoms associated with ASD.

Symptoms	Available medications
Behavioral symptoms, restlessness, self-aggression	Antipsychotics, Anticonvulsants
Socialization problems	Oxytocin, D-cycloserine, Memantine (experimental)
Sleep disorders	Melatonin, Antipsychotics, Antihistamines
Attention deficit hyperactivity disorder (ADHD)	Atomoxetine, Methylphenidate, Amphetamines, Desmethylamphetamines, Guanfacine
Tics	Antipsychotics, α2 agonists, SSRIs
Depression	SSRIs, SNRIs + Antipsychotics
Anxiety and OCD	SSRIs (high doses), Pregabalin
Psychosis	Antipsychotics

Adapted from Popow et al. (24).

Medications administered for psychiatric symptoms work by reducing or preventing abnormal electrical activity in the brain that leads to seizures. Currently, there are different drugs available, and their selection depends on the type of seizure, the child's age, and comorbid conditions (32–35). In cases where a person with ASD does not experience seizures, the administration of medications to address psychiatric symptoms will focus on addressing behavioral, emotional, or cognitive aspects associated with ASD. These medications may be prescribed to manage issues such as anxiety, hyperactivity, aggression, sleep disorders, or other psychiatric symptoms that may arise in individuals with ASD. The choice of medication will depend on the specific nature of the symptoms and the assessment by the healthcare professional. Medications administered for psychiatric symptoms associated with ASD are often used in combination with other types of medications such as anxiolytics, antipsychotics, and antidepressants, depending on the epileptic symptoms and the severity of the epilepsy episodes the person is experiencing (35). Table 3 details the main drugs for treating epilepsy based on the symptoms presented by individuals with ASD and epilepsy.

**Table 3:** Preferred pharmacological treatment for addressing epilepsy in the pediatric population with ASD.

Drug	Adverse effect	Indicated for epilepsy type
Carbamazepine	Sedation, drowsiness, anxiety, visuomotor incoordination, attention deficit, hyperactivity, behavioral disorders, or learning problems (27–30, 33–36)	Focal and focal to bilateral tonic clonic
Sodium valproate	Drowsiness, irritability, sleep disturbance, or motor incoordination (26–32)	Focal, focal to bilateral tonic clonic, generalized tonic clonic, and myoclonic

Another significant challenge is the high interaction among the drugs described above (26–37). Antipsychotic drugs have significant reactions and drug interactions with anticonvulsants and mood stabilizers, including carbamazepine, though not all of these interactions occur negatively (38, 39). Identifying the consequences of such combinations is highly relevant, as, in some cases, serious adverse reactions may develop. This is exemplified by De León et al.'s (40) of interactions between anti-seizure medicines (ASMs) and second-generation antipsychotics potentially leading to exceptional cases of pancreatitis, agranulocytosis/leukopenia, and heatstroke. Additionally, Hitchings (41), in a review study, suggests that antipsychotics, by reducing the antiepileptic effect of these drugs, may reduce the efficacy of anti-seizure drugs.

Clozapine, known for reducing the seizure threshold, is linked to dose-dependent electroencephalographic alterations, impacting approximately 3%–6% of patients under clozapine treatment.

Asenjo Lobos et al. (42) highlight favorable outcomes in combined therapy involving antipsychotics for patients exhibiting partial response, as evidenced in several double-blind studies. The combinations encompass olanzapine paired with lithium or valproate, risperidone combined with lithium or valproate, haloperidol alongside lithium or valproate, and quetiapine in conjunction with lithium or valproate.

Similarly, the effectiveness of combined therapy has been demonstrated in open-label studies for patients with both ASD and epilepsy showing partial response. Such studies endorse combinations like olanzapine with lithium, valproate, or carbamazepine; risperidone paired with lithium or valproate, and quetiapine combined with lithium or valproate (43). The simultaneous administration of these medications should be approached with caution due to the potential emergence of toxic symptoms at high doses. Moreover, acute manic symptoms, extrapyramidal effects, physiological disorders, or brain damage could manifest, altering the neurological structure in which the epilepsy focus is located (44–46).

## 3 Surgical treatment in the pediatric population with ASD and epilepsy

Medical treatments for ASD and epilepsy typically focus on managing the core symptoms of both disorders (ASD and epilepsy), addressing concurrent physical conditions such as sleep disorders, gastrointestinal issues, and sensory difficulties (47). However, unresolved issues persist in understanding the comorbidity between ASD and epilepsy. One fundamental challenge lies in the relationship between seizures and the central social symptoms of ASD. Although there is a high prevalence of epilepsy in individuals with ASD, the exact nature of how seizures contribute to the social aspects of ASD remains an actively researched area. Significant variability in the frequency of seizures also raises questions. While some children with ASD and epilepsy experience recurrent episodes, others may have a single episode in their lifetime. This variability not only complicates the understanding of the relationship between seizures and ASD but also presents challenges in identifying predictive patterns and personalized treatment strategies. Epilepsy itself represents a significant clinical problem, but the decision to resort to surgery must be approached with caution and is reserved for cases of refractory epilepsy with clearly defined cortical abnormalities that require surgical intervention (48). This decision entails the need for an accurate diagnosis and the identification of suitable candidates for surgical procedures, which remains an area in development. The comorbidity between ASD and epilepsy remains an unresolved clinical issue, exacerbated by a substantial lack of data and the complexity of interactions between both disorders (49). Ongoing research is essential to unravel the underlying mechanisms, better understand the variability in clinical presentation, and develop more effective and personalized treatment approaches for those affected by this comorbidity (50).

Upon receiving a referral, it is customary for the patient to undergo a thorough preoperative evaluation with the aim of identifying the epileptogenic zone and evaluating the patient's suitability for surgery (51). This evaluation typically includes a detailed medical history (analyzing clinical semiology, seizure frequency, severity, and prior treatments), video electroencephalography (EEG), magnetic resonance imaging (MRI) using an epilepsy protocol, and neuropsychological testing (52). Depending on the clinical context, additional tests such as positron emission tomography, single-photon emission computed tomography, magnetoencephalography, functional magnetic resonance imaging, and Wada testing may be considered (52, 53). The results of these assessments are then reviewed by a multidisciplinary team, which provides treatment recommendations. In certain cases, supplementary information is gathered through the surgical placement of intracranial electrodes to enhance the localization of the epileptogenic focus and identify critical brain areas for preservation during surgery (53).

For focal-onset seizures that can be localized in a surgically manageable area, available options encompass lesionectomy, temporal lobectomy, or extratemporal cortical resection. These procedures may be executed as a single intervention or in two stages, occasionally preceded by invasive EEG monitoring (54). In more severe epilepsies, alternatives may include functional disconnection (hemispherotomy) or anatomical hemispherectomy (complete removal of a hemisphere) (53, 54). Patients experiencing drop seizures, ineligible for resective options, may be candidates for palliative disconnection through a callosotomy of the corpus callosum. Palliative interventions like vagus nerve stimulation (VNS), and more recent approaches such as responsive neurostimulation (RNS) and deep brain stimulation (DBS), are also viable for patients not suitable for resection (55). Lastly, advancing technologies like laser ablation and gamma knife radiosurgery (GK) are continuously emerging, fostering ongoing exploration of minimally invasive approaches to addressing epileptogenic lesions (55, 56).

### 3.1 Surgical resective techniques for epilepsy

Lesionectomy stands out as a viable choice for addressing diverse lesions that provoke seizures, including neoplasms (such as gangliogliomas, oligodendrogliomas, astrocytomas, and dysembryonic neuroepithelial tumors) and vascular malformations (cavernomas and arteriovenous malformations) (57–59). In situations where there is alignment among seizure semiology, EEG monitoring findings, and lesion localization on magnetic resonance imaging (MRI), lesionectomy without the need for invasive monitoring is regarded as a favorable option (55–59). While the ultimate objective is complete lesion removal, this can prove challenging in eloquent regions. In many instances, the resection is confined to a portion of the lesion, such as retaining surrounding hemosiderin in the case of cavernomas and arteriovenous malformations (60).

Results after lesionectomy are generally excellent, particularly in pediatric patients. The extent of resection is critical, as total resection has been positively correlated with seizure freedom, reaching approximately 80%, while subtotal resection has a success rate of around 50%. Intraoperative electrocorticography guides resection and often results in the removal of perilesional tissue, with the potential to improve long-term seizure freedom rates (60, 61).

As for temporal lobectomy, one of the most studied surgical options, randomized controlled trials have shown that 58% of patients are seizure-free one year after surgery, compared to only 8% with medical management. The complication rate is relatively low, although side effects such as contralateral upper quadrantanopsia, loss of verbal memory, and, rarely, depression may arise (60–62). The success rate of temporal lobectomy in children is reported to be around 76% (62).

Extratemporal resection typically requires prior implantation of intracranial electrodes to map the epileptogenic focus and preserve eloquent brain areas. Although the effectiveness of extratemporal resection is lower than that of temporal foci, it significantly surpasses continuous medical management, with seizure freedom rates close to 56% in pediatric patients, according to a meta-analysis. Variability in results is associated with seizure duration, the presence of epileptogenic lesions, and/or partial seizures (63).

In extreme cases of catastrophic epilepsy in children and adolescents, such as hemimegalencephaly, hemispherectomy is presented as an option. This intervention aims to functionally disconnect or, in more aggressive cases, physically remove an affected cerebral hemisphere (64). “Hemispherotomy,” a less resective variant, has demonstrated similar success rates, with an overall seizure freedom rate of 73.4%. Functional disconnection, known as “disconnective hemispherectomy,” reduces intraoperative blood loss and minimizes anatomical resection, albeit with slightly higher risks of hydrocephalus (14%) and superficial hemosiderosis (65). Other less resective modifications have emerged under the term “hemispherotomy.” It is emphasized that up to approximately 14 years of age, pediatric and adolescent patients experience greater brain plasticity, contributing to substantially higher neurological recovery compared to adults undergoing more aggressive surgical interventions (64–66). Systematic studies have reviewed various hemispherectomy techniques in the pediatric/adolescent population, reporting an overall seizure freedom rate of 73.4%, with no significant differences in outcomes based on the type of hemispherectomy. Hemispherotomies, however, are associated with a lower risk of complications compared to other techniques. Additional research supports similar outcomes, highlighting seizure freedom rates of 63% or higher in patients five years post-surgical intervention (67).

### 3.2 Palliative procedures

Callosotomy is frequently reserved as a surgical intervention for managing medically resistant atonic seizures. Its primary objective is to reduce the occurrence of drop attacks, subsequently minimizing injuries resulting from falls. In contrast to procedures aiming for complete seizure freedom, callosotomy is considered a palliative measure (60–62).

Within this intricate landscape, the decision-making process between opting for a partial callosotomy, involving the anterior two-thirds of the corpus callosum, and a complete callosotomy remains a nuanced endeavor without a definitive consensus. The pursuit of superior seizure control leans decisively towards a complete callosotomy, boasting a remarkable 91% achievement of Engel class I–III, in stark comparison to the relatively lower 75% observed in partial callosotomies (59–61). The inclination towards the latter procedure may stem from a strategic intent to mitigate the occurrence of disconnection syndromes. While transient acute disconnection syndromes find a place in relative commonality, the occurrence of permanent disconnection syndromes is a rare phenomenon. Furthermore, strategic measures such as restricting the extent of callosal transection and orchestrating the procedure at a younger age or in staged interventions appear promising avenues to curtail the risk of disconnection syndromes (64).

On a divergent note, Vagus Nerve Stimulation (VNS), having received FDA approval in 1997, emerges as a compelling alternative for grappling with intractable partial-onset epilepsy, particularly in patients aged 12 and older. Despite the enigma surrounding its mechanism of action, VNS is postulated to exert its effects through thalamocortical projections. While the surgical complications of VNS are notably infrequent, boasting an infection rate of 4%–6%, and accompanied by generally well-tolerated side effects like hoarseness and voice changes, the occurrence of asystole is an exceedingly rare event, reported in less than 0.1% of patients. A recent meta-analysis paints a compelling picture, revealing a 50% reduction rate in seizures for half of the patients undergoing VNS therapy. Notably, this therapeutic avenue extends its benefits to both pediatric and adult populations with generalized epilepsy, despite its off-label application in both cohorts (58, 64).

In tandem with VNS, Responsive Neurostimulation (RNS) emerges as a notable player, securing FDA approval in 2013. Current indications cast a broad net, encompassing individuals aged 18 or older who grapple with at least three partial-onset seizures monthly, have experienced treatment failures, and have undergone comprehensive diagnostic tests to pinpoint a seizure focus amenable to stimulation (65). While the current approval lends credence to its clinical utility, ongoing research endeavors strive to illuminate its long-term efficacy, particularly in the context of treating medically resistant epilepsies in the pediatric population. A recent multicenter, double-blind, randomized controlled trial involving 191 patients paints an optimistic picture, indicating a median reduction of 44% in seizures at 1 year and an even more encouraging 53% at the 2-year mark within an open-label treatment interval (68). These strides in neuromodulatory therapies underscore a continuously evolving landscape, offering renewed hope and options for patients ensnared by the clutches of treatment-resistant epilepsy.

Finally, the precise mechanism by which Deep Brain Stimulation (DBS) exerts its anticonvulsant effect is not precisely known, but the most widely accepted explanation is that it induces an acute electrical disruption of synchronous activity at its origin, necessary for the propagation of ictal activity (48). It is noteworthy that, in DBS, most efferents of a target can follow action potentials at the stimulation frequency (49, 50). However, the generation of action potentials may be interfered with by the stimulation of afferent fibers towards the dendrites and somas of the target neurons, which can be excitatory or inhibitory, affecting the soma (49, 50). Nevertheless, the palliative option of deep brain stimulation for patients with poorly localized focal or generalized-onset epilepsy has been proposed for analysis, considering it as an emerging treatment in pediatrics, although it lacks FDA approval (49, 61).

### 3.3 Emerging minimally invasive therapies

In the realm of cutting-edge therapeutic approaches, interstitial laser thermal therapy guided by MRI emerges as a promising, less invasive method for ablating epileptic foci. This innovative procedure involves the meticulous placement of an optical fiber catheter, encased in a cooling sheath, within an identified epileptogenic focus. The subsequent application of laser energy orchestrates thermal ablation in the region of interest, unfolding a novel frontier in epilepsy treatment (67). Executed within the confines of the MRI suite, this procedure leverages real-time thermal imaging to meticulously monitor the treatment's progression, estimating the size and final location of the lesion with unparalleled precision. Currently, the narrative of its application in epilepsy treatment is predominantly woven through the fabric of case reports and small case series (68–70). The roster of treated lesions encompasses periventricular heterotopias, hypothalamic hamartomas, cortical dysplasia, and tubers. A groundbreaking study featuring 268 consecutively treated patients with Magnetic Resonance-guided Laser Interstitial Thermal Therapy (MRgLITT) in the medial temporal lobe has unveiled compelling rates of seizure freedom over time: 55.8% at 1 year, 52.5% at 2 years, and 49.3% at the last follow-up of ≥1 year (median of 47 months). Further bolstering these findings, combined results of Engel I or II showcase encouraging rates of 74.2% at 1 year, 75.0% at 2 years, and 66.0% at the last follow-up. Notably, an independent association has been discerned between preoperative focal to bilateral tonic-clonic seizures and the recurrence of seizures. In a striking turn of events, among patients grappling with seizure recurrence, a significant cohort attained seizure freedom post subsequent surgeries—be it anterior temporal lobectomy (ATL) or a repeat of MRgLITT—underscoring the profound effectiveness of these supplementary approaches in the realm of long-term seizure control (71).

Turning the narrative lens to another groundbreaking frontier, Gamma Knife (GK) radiosurgery has ascended in recent years as a precision-driven, stereotactic powerhouse for delivering focal radiation to intracranial epileptogenic targets, all while sparing surrounding tissues from appreciable radiation damage (72). This sophisticated tool offers a ray of hope in the treatment of epilepsy, presenting the capability to administer targeted radiation doses to specific regions of the affected brain. By elegantly sidestepping the need for whole-brain irradiation, the GK minimizes the collateral damage and toxicity associated with conventional radiotherapy. Its prowess in delivering focused and precise treatments positions it as a valuable asset in the nuanced landscape of epilepsy management, particularly when pharmacological avenues have proven futile. Beyond the realm of epilepsy, this technology currently finds widespread application in focal radiosurgical ablation, ranging from intracranial metastatic diseases and pituitary lesions to acoustic neuromas, refractory trigeminal neuralgia, and various other neurosurgical conditions (73). Its efficacy has been scrutinized in the context of mesial temporal lobe epilepsy, particularly when there is telltale evidence of hippocampal sclerosis in magnetic resonance imaging—a scenario traditionally addressed through selective amygdalohippocampectomy (74). For those navigating the treacherous waters of surgical risks or having faced disappointments in prior surgical interventions for epilepsy, Gamma Knife radiosurgery emerges as a beacon of hope—a non-invasive alternative boasting successful long-term seizure freedom outcomes of 60% or more (75–77). However, the saga continues with an earnest call for further research and clinical studies to meticulously evaluate the effectiveness and potential enduring benefits of stereotactic radiosurgery, notably the kind bestowed by the Gamma Knife, in the expansive tapestry of epilepsy treatment. This quest extends to comparing its outcomes with other therapeutic modalities, charting the course for the future of epilepsy care.

## 4 Discussion

This opinion article aimed to describe and analyze the most effective pharmacological and surgical treatments for addressing the symptoms in the pediatric population with ASD and epilepsy.

The analysis conducted in this study identified the challenge of treating the clinical picture experienced by individuals with ASD and epilepsy. Approaching this comorbidity from a pharmacological perspective is a complex task. Firstly, pharmacological treatment does not allow for the elimination of the aversive symptoms of ASD and epilepsy; instead, it focuses on alleviating and reducing the patient's symptoms. Furthermore, the heterogeneity of symptoms in individuals with ASD and epilepsy significantly complicates the selection of combined drugs to reduce behavioral and physiological symptoms of ASD, as well as epilepsy episodes. Additionally, drug interactions contribute to unwanted effects in patients with this comorbidity.

Secondly, medical interventions focus on alleviating symptoms by addressing concurrent physical conditions. For those resistant to pharmacotherapy, surgery, such as temporal resection or functional disconnection, is considered effective, with careful attention to thorough preoperative evaluation. Additionally, palliative procedures like callosotomy and neuromodulatory therapies such as VNS, RNS, and DBS offer promising treatments for patients who are not resection' candidates. Emerging therapies, like laser ablation and GK, present minimally invasive approaches. These surgical and therapeutic options constitute comprehensive strategies to address the complexity of the comorbidity between ASD and epilepsy, emphasizing the need to consider approaches beyond conventional medical options to enhance the quality of life for patients.

Current scientific literature does not present significant results that allow determining the most effective treatment choice for the pediatric population with ASD and epilepsy (37–42, 44, 53, 55–57). In this regard, (I) the clinical, genetic, and physiopathological heterogeneity, the multiple possible targets, and the different pathways involved in the pathogenesis; (II) the lack of case-control and cohort follow-up studies from the onset of symptoms in preschoolers (2 years or less) that differentiate between the natural evolution of the condition and the evolution of the condition with intervention; (III) the difficulty in conducting studies with patient and family consent; (IV) the absence of biomarkers that allow grouping similar patients with more homogeneous, evaluable, and reproducible clinical characteristics; (V) the lack of randomized, placebo-controlled, and double-blind studies; (VI) the difficulty in determining the duration of studies, as the evolution of children with ASD and epilepsy undergoes modifications not only attributable to pharmacological interventions but also inherent to development; and (VI) the lack of family adherence to research significantly limit the treatment plan for individuals with this comorbidity (11–14).

In conclusion, the treatment of children with ASD and epilepsy requires an interdisciplinary approach that includes rehabilitation, pharmacology, and ultimately, surgical intervention (23). It is important to mention that these treatments can be costly and may not always be available to all children with ASD and epilepsy (2–5). Accessibility to treatments is a significant concern for parents, caregivers, and healthcare professionals (6), which can greatly influence the choice of the most suitable treatment modality (24). Additionally, pharmacological, and surgical treatments may have side effects, emphasizing the importance of closely monitoring children during treatment to minimize them as much as possible (37). Finally, it is essential to consider the warning signs of ASD and epilepsy to provide early attention to comorbidity, allowing for a differential diagnosis and designing evidence-based personalized interventions to enhance children's development and ensure an improvement in their quality of life (13).
